# High-Efficiency Adsorption of Methylene Blue by Balsa Wood Waste-Based Microporous Carbon

**DOI:** 10.3390/molecules31081251

**Published:** 2026-04-09

**Authors:** Yuzhou Zhou, Lan Geng, Leihui Zhang, Yong Su, Rui Liu, Fang Guo, Limin Zhang

**Affiliations:** 1 College of Resource, Hunan Agricultural University, Changsha 410128, China; 18207429640@163.com (Y.Z.);; 2 School of Resources and Environment, Hunan University of Technology and Business, Changsha 410083, China; 3 China Nonferrous Metal Mining (Group) Co., Ltd., Beijing 100029, China; 4 Institute of Subtropical Agriculture, Chinese Academy of Sciences and Changsha Research Station for Agricultural & Environmental Monitoring, Changsha 410125, China

**Keywords:** *Ochroma pyramidale*, super activated carbon, ultra-high specific surface area, methylene blue, adsorption performance

## Abstract

Biomass-based adsorbents for methylene blue (MB) currently face critical bottlenecks including raw material homogenization, insufficient adsorption capacity, and an unclear structure–activity relationship. To address these limitations, we prepared porous super activated carbon (SAC) with ultra-high specific surface area via KOH activation, using industrial balsa wood (*Ochroma pyramidale*) waste from the wind power industry as the precursor. The adsorption behavior and underlying mechanism of the as-prepared SAC towards MB were systematically investigated. The as-prepared SAC has an ultra-high specific surface area of 3833 m^2^/g, with a well-developed microporous structure matching the molecular size of MB. It exhibited a maximum monolayer MB adsorption capacity of 1037.76 mg/g, superior to similar biomass-based materials. Near-complete removal of high-concentration MB was achieved at an SAC dosage of 0.4 g/L, and the material maintained stable performance across a wide pH range of 4 to 10. The adsorption of MB onto SAC fitted well with the Langmuir isotherm and pseudo-second-order kinetic models, dominated by monolayer physisorption. The outstanding adsorption performance originated from the synergistic contribution of the pore confinement effect, π-π conjugation, electrostatic interaction, and hydrogen bonding. This work provides a new strategy for high-value utilization of balsa wood industrial waste and efficient treatment of dye wastewater.

## 1. Introduction

With the rapid development of the textile and dyeing industries, dye wastewater has become one of the primary sources of organic pollution in the global aquatic environment. Statistics show that the annual global production of dyes exceeds 750,000 tons, among which 10–15% is directly discharged into water bodies without treatment during production and application, resulting in severe chromaticity pollution and ecotoxicity [1,2]. Methylene blue (MB), as a typical cationic phenothiazine dye, is widely used in dyeing, pharmaceutical, chemical indicator and other fields. It is characterized by recalcitrance to biodegradation, high toxicity, and easy bioaccumulation. Even at a concentration below 1 mg/L in water, MB can induce oxidative stress and genotoxicity to aquatic organisms, and ultimately endanger human health through the food chain [3]. Therefore, the development of efficient and low-cost technologies for the advanced treatment of MB-containing wastewater has become an urgent demand in the field of water environment remediation.

Among numerous wastewater treatment technologies, adsorption technology is recognized as one of the most promising technologies for the industrial treatment of dye wastewater due to its advantages of simple operation, high treatment efficiency, lack of secondary pollution, and wide applicable concentration range [4]. As the most widely used adsorbent, activated carbon exhibits excellent adsorption performance for various organic pollutants, which is attributed to its well-developed pore structure, abundant surface functional groups, and outstanding chemical stability. However, traditional commercial activated carbon is mostly produced from coal and virgin wood, which has the drawbacks of non-renewable raw materials, high cost, and specific surface area generally lower than 2000 m^2^/g, limiting its large-scale application in the treatment of high-concentration dye wastewater [5]. In recent years, the preparation of biomass-based activated carbon using industrial and agricultural biomass solid waste as precursors has become a research hotspot in the field of adsorption materials, owing to its dual environmental benefits of solid waste reduction and pollution control [6,7]. At present, agricultural solid wastes such as coconut shells, walnut shells, straw, and bamboo sawdust have been widely used to prepare activated carbon for MB adsorption. Nevertheless, existing studies still have three key bottlenecks. First, there is serious homogenization of raw materials. There are few studies on the high-value and targeted functional utilization of bulk industrial balsa wood waste generated from high-end manufacturing industries such as wind power and aerospace. At present, most of this waste is disposed of by landfill and incineration, which not only causes serious resource waste but also brings air and soil pollution. Second, the preparation process lacks precise regulation. The obtained biochar generally has the problems of insufficient specific surface area, poor micropore development, and mismatch between pore structure and dye molecular size, resulting in mostly low MB adsorption capacity, which is difficult to meet the practical treatment requirements [8,9].

Notably, balsa wood (*Ochroma pyramidale*) is one of the fastest-growing commercial woods in the world. Due to its ultra-low density and excellent mechanical properties, it is widely used in wind turbine blades, aerospace structural sandwich panels and other fields. The annual output of balsa wood processing waste from the wind power industry in China alone exceeds 100,000 tons. Balsa wood inherently has a natural porous structure and high carbon content, making it an ideal precursor for the preparation of porous carbon with ultra-high specific surface area [10,11]. However, there are few studies on MB adsorption using biochar derived from virgin balsa wood as the matrix. On this basis, this study used industrial balsa wood processing waste as the precursor to controllably prepare microporous super activated carbon (SAC) with ultra-high specific surface area via the KOH activation method. The pore structure and surface chemical properties of the material were clarified through multi-scale characterization. The effects of adsorbent dosage and solution pH on the MB adsorption performance of SAC were systematically investigated. Combined with isotherm and kinetic model fitting, the equilibrium characteristics and kinetic behavior of the adsorption process were analyzed. Finally, a complete structure–activity relationship of “raw material-structure-surface property-adsorption performance” was constructed, and the underlying mechanism of the efficient adsorption was elucidated.

## 2. Results and Discussion

### 2.1. Structural and Surface Chemical Characteristics of the As-Prepared SAC

**Figure 1:**
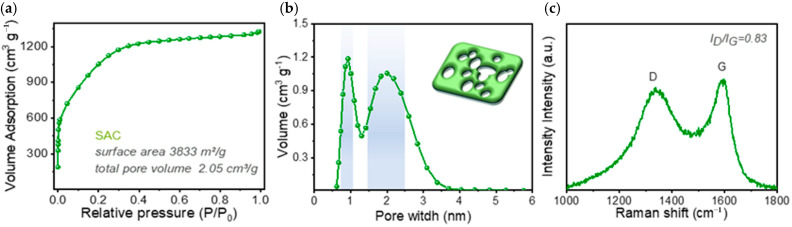
(**a**) Nitrogen adsorption–desorption isotherm of the as-prepared SAC; (**b**) Pore size distribution curve of the as-prepared SAC; (**c**) Raman spectrum of the as-prepared SAC.

Pore structure is the core structural determinant of the adsorption performance of activated carbon. In this study, the specific surface area of the biochar was precisely regulated via the KOH activation process to achieve the controllable preparation of a microporous structure highly matched with the molecular size of MB. Figure 1a presents the nitrogen adsorption–desorption isotherm of the as-prepared SAC, which is a typical Type I isotherm (per IUPAC classification). In the low relative pressure range of P/P0 < 0.1, the nitrogen adsorption capacity increased sharply, and then gradually leveled off with the increase in relative pressure. Meanwhile, no obvious hysteresis loop was observed in the isotherm, indicating that the material was dominated by a microporous structure [12].

Calculation results showed that the as-prepared SAC had an ultra-high specific surface area of 3833 m^2^/g, with a total pore volume of 2.05 cm^3^/g, among which the micropore volume reached 1.94 cm^3^/g. This specific surface area was higher than that of previously reported biomass-based activated carbons derived from bamboo, coconut shell, straw and other feedstocks (generally < 2000 m^2^/g), and also outperformed the specific surface area level of most existing balsa wood-based adsorbents [13,14]. As can be seen from the pore size distribution curve in Figure 1b, the pore size of SAC was mainly concentrated in the range of 0.6–2 nm, with an average pore size of 2.14 nm, and the micropore volume accounted for more than 95% of the total pore volume. The pore size of SAC was highly matched with the kinetic diameter of MB molecules (approximately 1.3 nm), which not only provided massive active sites for the adsorption of MB molecules, but also enhanced the interaction between MB molecules and the pore wall through the significant pore confinement effect, laying a core structural foundation for the subsequent achievement of ultra-high adsorption capacity [15].

Typical D band and G band were observed in the Raman spectrum of SAC (Figure 1c), located near 1350 cm^−1^ and 1580 cm^−1^, respectively. After baseline subtraction, the net intensities of the two bands were 2137 and 2569, respectively, corresponding to an ID/IG ratio of 0.83. This ratio is lower than that of most amorphous biomass-based activated carbons [5], indicating that the as-prepared SAC retains a more ordered graphitic microcrystalline structure while introducing abundant structural defects and disordered carbon regions. These defect sites can serve as adsorption active centers, and the ordered graphitized carbon domains can form π-π stacking interactions with the aromatic ring structure of dye molecules, which is conducive to the adsorption of MB. No obvious 2D band was detected in the spectrum, further confirming that the material was dominated by amorphous carbon.

**Figure 2:**
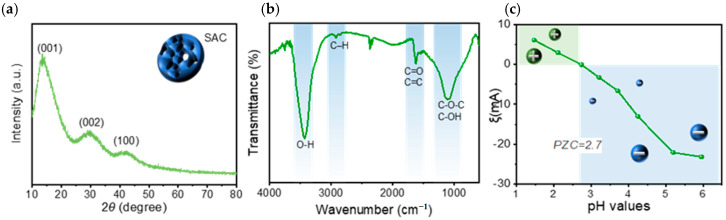
(**a**) XRD pattern of the as-prepared SAC; (**b**) FTIR spectrum of the as-prepared SAC; (**c**) Zeta potential curve of the as-prepared SAC as a function of pH.

Figure 2a shows the XRD pattern of the as-prepared SAC. A broad diffraction hump appears in the 2θ range of 20° to 30°, which is attributed to the (002) crystal plane of carbon materials. The peak position is close to 30°, indicating that the SAC has a tight interlayer spacing and a locally compact graphitic stacking structure, while the broad peak shape confirms that the material is dominated by amorphous carbon with partial ordered microcrystalline domains, which is consistent with the Raman characterization results.

In addition, as shown in Figure 2b, the FTIR spectrum of SAC exhibits a broad and strong absorption band in the range of 3700 to 3000 cm^−1^, centered at approximately 3400 cm^−1^, which is attributed to the O-H stretching vibration of hydroxyl groups with distinct hydrogen bond association characteristics. The weak absorption peaks at 2920 cm^−1^ and 2850 cm^−1^ correspond to the aliphatic C–H stretching vibration. The absorption peak near 1600 cm^−1^ is assigned to the superposition of the C=O stretching vibration of carboxyl or carbonyl groups and the C=C skeleton vibration of aromatic rings, which directly verifies the existence of unsaturated carbon structures derived from a small amount of graphitized microcrystals on the material surface, this delocalized π bonds of the carbon skeleton can form stable π-π conjugation interactions with the π electrons in the benzene rings and phenothiazine ring of MB molecules [16]. The absorption peak near 1400 cm^−1^ corresponds to the O-H bending vibration of phenolic hydroxyl or carboxyl groups and the C-H bending vibration. The strong absorption peak at 1100 cm^−1^ is assigned to the stretching vibration of the ether bond (C-O-C) and C-OH groups, indicating the presence of oxygen-containing bridging structures. The absorptions in the fingerprint region below 1000 cm^−1^ are related to the out-of-plane bending vibration of aromatic C-H bonds.

Therefore, the abundant oxygen-containing functional groups such as hydroxyl and carboxyl groups on the SAC surface can form stable hydrogen bonding interactions with the amino groups and heterocyclic nitrogen atoms in MB molecules. Meanwhile, the π-π conjugation structure also makes a critical contribution to MB adsorption. Furthermore, as shown in Figure 2c, the curve of the Zeta potential of SAC as a function of pH indicates that the isoelectric point of SAC is 2.7, and the potential decreases gradually with increasing pH. When the solution pH is higher than 2.7, the material surface is negatively charged. With the rise in pH, the oxygen-containing functional groups such as carboxyl and phenolic hydroxyl groups on the surface undergo deprotonation, leading to a gradual increase in the negative charge density on the surface. MB exists in the form of cations in aqueous solution, so under the condition of pH > 2.7, a strong electrostatic attraction occurs between the negatively charged SAC surface and the positively charged MB molecules, which provides the important driving force for the rapid adsorption of MB [17].

**Figure 3:**
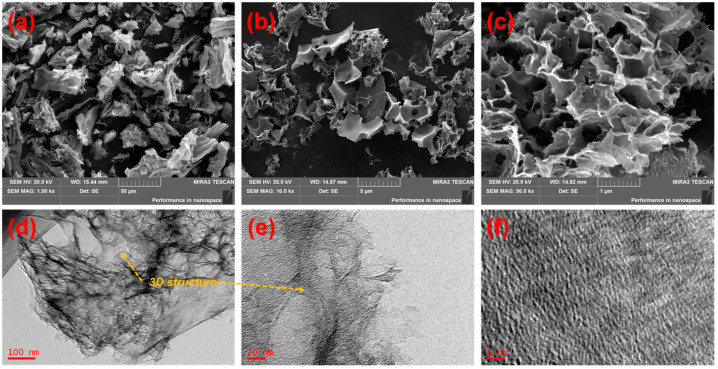
(**a**–**c**) SEM images of the as-prepared SAC; (**d**–**f**) TEM images of the as-prepared SAC.

Figure 3 presents the SEM and TEM images of the as-prepared SAC. The SEM images in Figure 3a–c show that the activated SAC particles exhibit an irregular block-like morphology, with a particle size ranging from tens to hundreds of micrometers. The particle surface retains the inherent natural vessel structure of the raw balsa wood, forming an open and interconnected macropore network (pore size > 10 μm). These macropores can serve as channels for the rapid diffusion of molecules, effectively shortening the diffusion path of MB molecules from the bulk solution to the internal micropores. The high-magnification SEM images reveal that the interior of the activated carbon has a disordered sponge-like carbon skeleton with an overall low contrast, indicating the low density and highly developed porosity of the material, which is in good agreement with the XRD characterization results.

The TEM images in Figure 3d–f display that densely distributed micropores and ultramicropores exist inside the SAC, with pore sizes mainly concentrated in the range below 2 nm, and the pore walls are thin and interconnected. This structural feature directly accounts for the ultra-high specific surface area of 3833 m^2^/g and micropore volume of 1.94 cm^3^/g obtained from the nitrogen adsorption–desorption test. The abundant structural defects revealed by the Raman spectrum are manifested as unsaturated carbon atoms and disordered carbon layers at the edges of the micropores in the TEM images, which provide more active sites for adsorption. Combined with the SEM and TEM observations, the activated carbon after pore-forming activation forms a hierarchical pore structure dominated by micropores and supplemented by macropores and mesopores, realizing the synergistic effect of macropore transport and micropore storage.

### 2.2. Effects of Adsorption Process Parameters on Adsorption Performance

**Figure 4:**
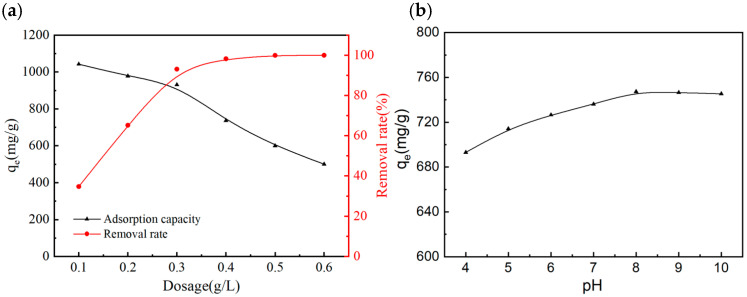
(**a**) Effects of the as-prepared SAC dosage on the adsorption capacity and removal rate of MB; (**b**) Effect of solution pH on the adsorption performance of the as-prepared SAC.

Figure 4a depicts the effects of SAC dosage on the adsorption capacity and removal rate of MB. As can be seen from the figure, at an initial MB concentration of 300 mg/L and pH = 7, as the SAC dosage increased from 0.1 g/L to 0.6 g/L, the MB removal rate continuously rose from 34.73% to nearly 99.12%, while the equilibrium adsorption capacity showed a consistent downward trend. This phenomenon was attributed to that the increase in adsorbent dosage provided more available adsorption active sites, thus driving the continuous increase in removal rate. However, at an excessively high dosage, agglomeration occurred between adsorbent particles, resulting in the encapsulation of partial active sites. Meanwhile, the limited number of MB molecules in the solution made the active sites of per unit mass of adsorbent unable to be fully utilized, hence the continuous decrease in adsorption capacity [18]. When the dosage was 0.4 g/L, the MB removal rate was close to 100%, and no significant enhancement in removal rate was observed with further increase in dosage. Therefore, 0.4 g/L was determined as the optimal dosage of the as-prepared SAC. Notably, this dosage was lower than that of previously reported biomass-based activated carbons (generally > 1 g/L), which means that in practical engineering applications, the required dosage of the SAC prepared in this study is less than 40% of that of traditional biochar for treating MB wastewater with the same concentration, which can effectively reduce the application cost of adsorbents.

Solution pH is a key factor affecting adsorption performance. It not only alters the surface charge properties of the adsorbent, but also affects the existing speciation of MB molecules in aqueous solution, which directly determines the applicability of the adsorbent in actual wastewater treatment. Figure 4b shows the effect of initial pH on the MB adsorption performance of SAC. As can be seen, at an initial MB concentration of 300 mg/L and SAC dosage of 0.4 g/L, the adsorption capacity of SAC for MB maintained a high level in the pH range of 4 to 10. As the pH increased from 4 to 8, the adsorption capacity rose slowly from 721.35 mg/g to 746.36 mg/g, and the adsorption capacity tended to be stable when pH exceeded 8. Combined with the zeta potential results, the SAC surface is negatively charged at pH values above 2.7, and the surface negative charge density increases slightly with the increase in pH, while the overall surface chemical properties of the material do not change significantly in this pH range. Therefore, the electrostatic attraction between SAC and positively charged MB molecules remains at a high level, leading to a slight increase in adsorption capacity with increasing pH [19]. Notably, electrostatic interaction was not the sole dominant mechanism for the adsorption. In addition, the van der Waals dispersion interactions between MB molecules and the carbon pore wall in the matched micropores are extremely insensitive to pH changes, which also provides a key contribution to the stable adsorption performance of SAC in a wide pH range. The pH of actual printing and dyeing wastewater usually fluctuates in the range of 6 to 9, while the SAC prepared in this study maintains outstanding adsorption performance in the wide pH range of 4 to 10. It can achieve efficient treatment without additional pH adjustment of the wastewater, which simplifies the practical treatment process and exhibits excellent engineering applicability.

### 2.3. Adsorption Isotherm Characteristics

**Figure 5:**
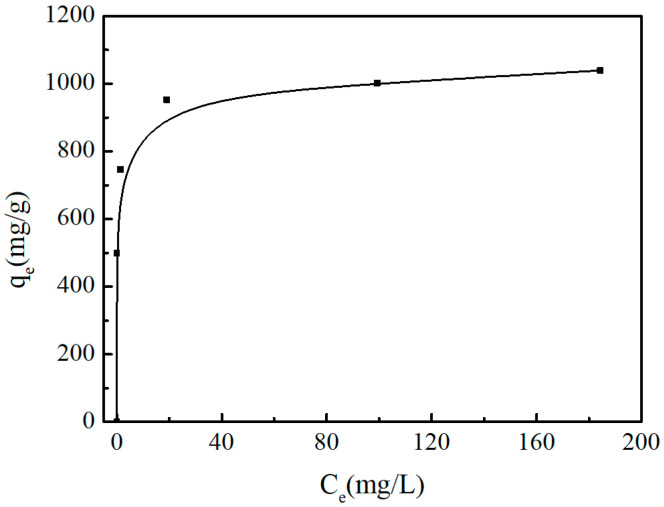
Adsorption equilibrium isotherm of MB onto the as-prepared SAC.

Adsorption isotherm can reflect the distribution behavior of adsorbate between the solid and liquid phases at adsorption equilibrium, and serves as the core basis for revealing the adsorption mechanism and evaluating the upper limit of adsorption performance. Figure S1 shows the effects of adsorption time and initial methylene blue (MB) concentration on the MB adsorption capacity of SAC at 30 °C. The experimental conditions are as follows: SAC dosage of 0.4 g/L, initial solution pH of 8, adsorption time ranging from 0 to 120 min, and initial MB concentration ranging from 200 mg/L to 600 mg/L. The results show that the equilibrium adsorption capacity of SAC for MB does not change significantly when the adsorption time is extended to 120 min, confirming that the adsorption reaction reaches equilibrium at 120 min. Figure 5 presents the adsorption equilibrium isotherm of methylene blue (MB) onto the as-prepared SAC under the same conditions. The results revealed that the equilibrium adsorption capacity increased sharply with the rise in MB concentration in the low concentration range, while the growth trend of adsorption capacity slowed down and gradually reached adsorption saturation in the high concentration range, indicating that the adsorption sites of the material were gradually occupied by MB molecules.

**Figure 6:**
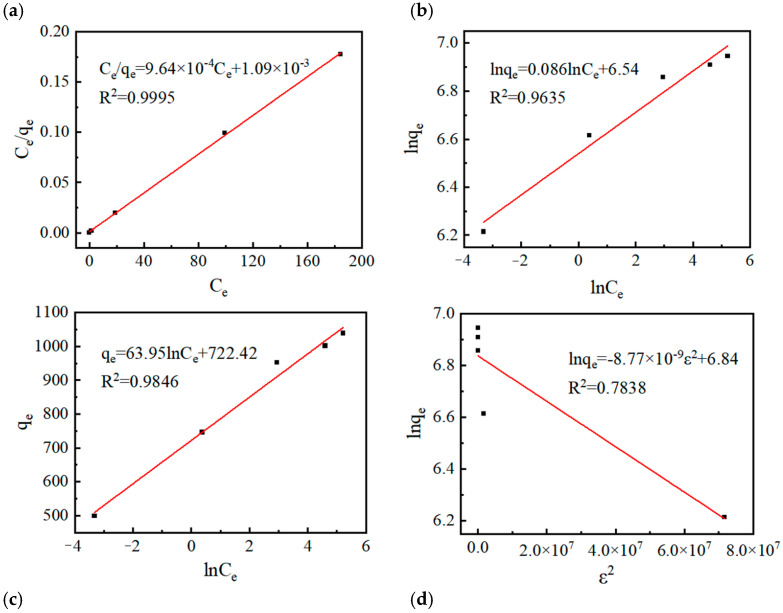
(**a**) Langmuir, (**b**) Freundlich, (**c**) Temkin, and (**d**) Dubinin-Radushkevich adsorption isotherms of methylene blue onto SAC at 30 °C.

Subsequently, four isotherm models, namely Langmuir, Freundlich, Temkin, and Dubinin-Radushkevich, were employed to fit the adsorption isotherm data in this study (Fitting methods: Supporting Information). The applicability of each isotherm equation was evaluated via the correlation coefficient (R^2^), and the fitting parameters are listed in Table 1. As shown by the fitting results (Figure 6), the correlation coefficient R^2^ of the Langmuir isotherm model reached as high as 0.9995, which was far superior to that of the other three models. This result indicated that the adsorption of MB onto the as-prepared SAC conformed well to the Langmuir isotherm model, that is, the adsorption process was dominated by the core characteristic of monolayer adsorption on homogeneous adsorption sites, with no significant interaction between adsorbed molecules. This also verified the high homogeneity of the adsorption sites on the SAC surface, which benefited from the precise regulation of the material surface structure by the activation process.

According to the fitting results of the Langmuir model, the maximum monolayer adsorption capacity (q_m_) of the as-prepared SAC towards MB reached 1037.76 mg/g. This value is significantly superior to the MB adsorption capacity of most wood-based and agricultural/forestry waste-derived activated carbons (Table 2), placing it among the leading levels of currently reported biomass-based adsorbents for MB adsorption.

### 2.4. Adsorption Kinetic Characteristics

To investigate the kinetic behavior and rate-controlling step of the adsorption process, the pseudo-first-order and pseudo-second-order kinetic models were employed in this study to fit the adsorption kinetic data at different initial MB concentrations (The Kinetic Adsorption equation: Supporting Information). The pseudo-second-order kinetic model is based on the assumption that the adsorption rate is governed by the surface adsorption interaction between adsorbate molecules and the active sites on the adsorbent surface.

**Figure 7:**
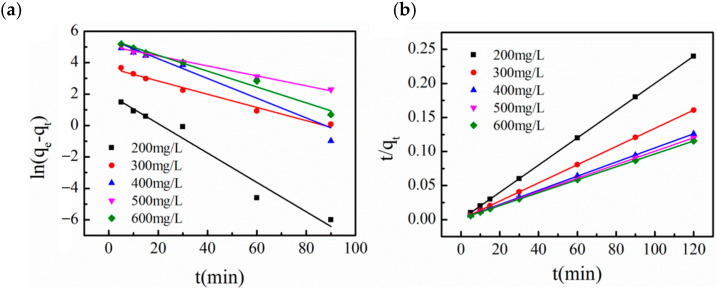
(**a**) Pseudo-first-order and (**b**) pseudo-second-order kinetics of methylene blue onto SAC at 30 °C.

For different initial MB concentrations ranging from 200 to 600 mg/L, lnq_e_−q_t_ was plotted against time t (Figure 7a), and the K_1_ values were calculated from the slope of the linear lines, with the results listed in Table 3. Although the correlation coefficients (R^2^) of the pseudo-first-order kinetic model at all initial concentrations were higher than 0.85, there was a large deviation between the experimentally measured q_e_ values and the model-calculated values, indicating that the adsorption process of MB onto the as-prepared SAC did not conform to the pseudo-first-order kinetic equation. For different initial concentrations, t/q_t_ was plotted against time t (Figure 7b), and the K_2_ values were calculated from the intercept of the linear lines, with the results listed in Table 3. At all initial concentrations, the correlation coefficients (R^2^) of the pseudo-second-order kinetic model were all higher than 0.99, and the experimentally measured q_e_ values were in excellent agreement with the model-calculated values, demonstrating that the adsorption process of MB onto the as-prepared SAC well followed the pseudo-second-order kinetic model.

As shown by the fitting results in Table 3, the correlation coefficients (R^2^) of the pseudo-second-order kinetic model were all ≥ 0.998 at all initial concentrations, and the theoretical equilibrium adsorption capacity (q_e,cal_) calculated by the model was in excellent agreement with the experimental value (q_e,exp_). In contrast, the pseudo-first-order kinetic model presented a poor fitting effect, with an extremely large deviation between the theoretical and experimental values. These results indicate that the adsorption process of MB onto the as-prepared SAC conforms well to the pseudo-second-order kinetic model, which means that the rate-determining step of the adsorption process is the surface adsorption interaction between MB molecules and the active sites on the SAC surface, rather than the intra-particle diffusion of MB molecules.

### 2.5. Analysis of Adsorption Structure–Activity Relationship and Interaction Mechanism

Combined with the pore structure characterization, surface chemical property analysis and adsorption experimental results, this study systematically constructed the adsorption structure–activity relationship of balsa wood-based SAC for MB, and elucidated the underlying mechanism of its efficient adsorption. It should be noted that the enthalpy of adsorption is the key quantitative indicator to distinguish physisorption and chemisorption. In this study, no chemical bond formation between MB and SAC was observed via FTIR characterization before and after adsorption, and all the interactions involved in the adsorption process (pore confinement, π-π conjugation, electrostatic interaction, hydrogen bonding) belong to the category of physisorption, which is consistent with the characteristics of monolayer adsorption fitted by the Langmuir model. Therefore, it is confirmed that the efficient adsorption of MB by the as-prepared SAC is a multi-mechanism synergistic process dominated by physisorption. The detailed mechanism is described as follows:

(1) Pore confinement effect is the structural basis for ultra-high adsorption capacity.

The as-prepared SAC has an ultra-high specific surface area of 3833 m^2^/g and a well-developed microporous structure. Its pore size (0.6–2 nm) is highly matched with the kinetic diameter of MB molecules (approximately 1.3 nm), which not only provides massive active sites for MB adsorption, but also enhances the van der Waals dispersion interactions between MB molecules and the pore wall via the pore confinement effect, thus greatly improving the adsorption binding energy. Notably, the dispersion interaction in the confined micropores is extremely insensitive to pH changes, which provides a key contribution to the stable adsorption performance of SAC in a wide pH range. This pore confinement effect is the core prerequisite for the SAC to achieve an ultra-high MB adsorption capacity of 1037.76 mg/g.

(2) π-π conjugation interaction is the core driving force for high-efficiency adsorption

Raman and XRD characterization results confirm that the as-prepared SAC retains an ordered graphitic microcrystalline structure while introducing abundant defect sites. The delocalized π bonds in the graphitized carbon skeleton can form stable π-π conjugation interactions with the π electrons in the benzene rings and phenothiazine ring of MB molecules, which further enhances the binding force between the adsorbent and MB molecules. This interaction belongs to physisorption, and is barely affected by solution pH, which is another important reason for the material to maintain stable adsorption performance in a wide pH range, and also the core factor for the material to achieve excellent adsorption performance for aromatic ring-containing dye molecules.

(3) Electrostatic interaction is an important driving force for rapid adsorption

The isoelectric point of SAC is 2.7. Within the pH range of actual printing and dyeing wastewater, the material surface is negatively charged, which generates strong electrostatic attraction with the positively charged MB cations in aqueous solution. This is also the core reason for the slightly better adsorption performance under weakly alkaline conditions and the stable adsorption effect of the material in a wide pH range.

(4) Hydrogen bonding interaction is an important supplement to adsorption performance

The abundant oxygen-containing functional groups such as hydroxyl and carboxyl groups on the SAC surface can form hydrogen bonding interactions with the amino groups and heterocyclic nitrogen atoms in MB molecules, further improving the adsorption binding capacity. Meanwhile, the abundant oxygen-containing functional groups enhance the dispersibility of the adsorbent in aqueous solution, improve the utilization rate of active sites, and further optimize the adsorption performance.

In summary, the efficient adsorption of MB by balsa wood-based SAC is a multi-mechanism synergistic process dominated by monolayer physisorption, which takes the pore confinement effect and π-π conjugation interaction as the core, and electrostatic interaction and hydrogen bonding interaction as important supplements. In this study, the pore structure and surface chemical properties of the material were regulated via the activation process to achieve the targeted optimization of MB adsorption performance, which provides a theoretical reference for the targeted design of similar biomass-based adsorption materials.

## 3. Materials and Methods

### 3.1. Experimental Materials and Reagents

Industrial balsa wood waste was obtained from industrial offcuts of a wind turbine blade processing factory in Anyang City, Henan Province, China. Methylene blue (methylene blue trihydrate, analytical grade, AR) was purchased from Sinopharm Chemical Reagent Co., Ltd. (Shanghai, China). Potassium hydroxide (KOH), hydrochloric acid (HCl), and sodium hydroxide (NaOH) were all of analytical grade and purchased from Tianjin Kemiou Chemical Reagent Co., Ltd. (Tianjin, China). Ultrapure water was used throughout all experiments in this study.

### 3.2. Preparation of Balsa Wood-Based Super Activated Carbon

The optimal preparation conditions were determined via single-factor optimization experiments as follows: an alkali-to-carbon ratio of 5:1, an activation temperature of 750 °C, and an activation time of 2 h. The preparation process under optimal conditions is described below.

First, balsa wood waste was repeatedly rinsed with ultrapure water to remove surface impurities, and then dried in a forced air drying oven for 24 h to constant weight. The dried material was pulverized by a high-speed pulverizer and sieved through a 100-mesh sieve (particle size < 0.15 mm) to obtain the balsa wood powder precursor. Subsequently, the balsa wood powder was placed in a tube furnace and carbonized at 500 °C for 2 h under a high-purity nitrogen atmosphere (flow rate: 100 mL/min) with a heating rate of 5 °C/min. The carbonized product was collected after naturally cooling to room temperature.

The carbonized product was mixed with KOH at an impregnation mass ratio of 1:5, and an appropriate amount of ultrapure water was added with stirring until KOH was completely dissolved. The mixture was impregnated at room temperature for 24 h to ensure sufficient contact between the activator and the carbon matrix. After impregnation, the mixture was dried in a vacuum drying oven at 100 °C for 24 h to remove moisture. The dried mixture was placed back into the tube furnace, heated to 300 °C at a heating rate of 5 °C/min under a nitrogen atmosphere, held for 30 min for dehydration, and then further heated to 750 °C for constant-temperature activation for 2 h.

After the product naturally cooled to room temperature, it was collected and rinsed with ultrapure water first to remove residual activator. Then, the product was oscillated and washed with 0.5 mol/L HCl solution at 80 °C for 2 h to remove soluble salts generated during the activation reaction. Finally, the product was repeatedly washed with hot ultrapure water until the pH of the filtrate was nearly neutral (pH = 6.5–7.5). After vacuum filtration and drying to constant weight, the balsa wood-based super activated carbon was obtained and labeled as SAC.

### 3.3. Material Characterization and Testing

The nitrogen adsorption–desorption isotherms of the samples were measured under 77 K liquid nitrogen conditions using an Autosorb-iQ automatic specific surface area and pore size analyzer (Quantachrome Instruments, Boynton Beach, FL, USA). The specific surface area was calculated via the Brunauer–Emmett–Teller (BET) model. The micropore size distribution was analyzed using the Horváth–Kawazoe (HK) model, and the mesopore size distribution and pore volume were analyzed via the Barrett–Joyner–Halenda (BJH) model, to clarify the pore structure characteristics of the materials. The microscopic morphology and pore channel structure of the samples were observed using a Nova NanoSEM 230 field emission scanning electron microscope (FE-SEM, FEI, Brno, Czech Republic) and a JEM-2100F field emission transmission electron microscope (FE-TEM, JEOL, Tokyo, Japan), to visually present the regulation effect of the activation process on the material structure. Zeta potential experiments were performed using a Malvern Zeta Sizer Nano Series instrument (Malvern Panalytical, Worcestershire, UK). All measurements were carried out at room temperature in a 0.01 M potassium nitrate electrolyte solution. A suspension containing 0.01 wt% SAC particles (100% passing through a 38 μm sieve) was prepared in the electrolyte solution. The pH of the suspension was adjusted to the required operating value by adding dilute HCl or NaOH solution. The final result was the average value of three independent measurements, with a typical variation of ±2 mV. The functional group structure on the sample surface was analyzed via a Tensor II Fourier transform infrared spectrometer (FT-IR, Bruker, Ettlingen, Germany) with a spectral range of 4000–400 cm^−1^, to clarify the surface chemical properties of the material and provide key evidence for the analysis of the adsorption mechanism. The graphitization degree of the samples was tested using a LabRAM HR Evolution Raman spectrometer (HORIBA, Villeneuve-d’Ascq, France) with a laser wavelength of 532 nm and a test range of 150–2000 cm^−1^. The crystal structure of the samples was analyzed using an Ultima IV X-ray diffractometer (XRD, Rigaku, Tokyo, Japan) with a scanning range of 5–80° and a scanning rate of 5°/min.

### 3.4. Batch Adsorption Experiments

Into a group of 150 mL Erlenmeyer flasks, 0.01–0.06 g of SAC and 100 mL of dye solution with a specified initial concentration (200–600 mg/L) were added respectively and mixed evenly. Subsequently, the initial pH of the mixed solution was adjusted to the set range (4–10) by dropwise addition of dilute HCl or NaOH solution. The Erlenmeyer flasks containing the adjusted suspension were placed in a constant temperature oscillator and shaken for 120 min at a constant rotation speed of 150 rpm and a constant temperature of 30 °C.

After reaching adsorption equilibrium, the Erlenmeyer flasks were taken out, and the mixed solution was filtered to obtain a clear methylene blue solution, followed by measurement of the final concentration of methylene blue in the solution. The equilibrium adsorption capacity q_e_ (mg/g) and the removal rate of methylene blue were calculated by the following formulas, respectively:

(1) $$q_{e}=\frac{(C_{0}-C_{e})V}{W}$$

(2) $$\mathrm{Removal}\;\mathrm{rate}=\frac{\;C_{0}\;{-\;C}_{e}}{C_{0}}\times 100\%$$

C_0_ and C_e_ (mg/L) are the initial and equilibrium concentrations of methylene blue, respectively; V is the volume of the methylene blue solution (L); W is the mass of the dried SAC added (g).

The operation procedure of batch kinetic experiments was basically consistent with that of the equilibrium experiments, except that aqueous samples were collected at preset time intervals. The concentration of methylene blue at time t was measured in the same way, and the adsorption capacity at time t q_t_ (mg/g) was calculated by the following formula:

(3) $$q_{t}=\frac{(C_{0}\;-{\;C}_{t})V}{W}$$

C_0_ and C_t_ (mg/L) are the concentrations of methylene blue in the aqueous solution at the initial time and time t, respectively; V is the volume of the methylene blue solution (L); W is the mass of the dried SAC added (g).

### 3.5. Determination of Methylene Blue Content

The concentration of methylene blue in the aqueous solution was measured using a UV-2600 UV-Vis spectrophotometer (Shimadzu Corporation, Kyoto, Japan) at a wavelength of 665 nm. The standard curve of methylene blue within the concentration range of this study had good repeatability, with a correlation coefficient (R^2^) of 0.9992, indicating a favorable linear relationship of the standard curve.

## 4. Conclusions

In this study, hierarchical porous super activated carbon with an ultra-high specific surface area of 3833 m^2^/g was successfully prepared via the KOH chemical activation method using bulk industrial balsa wood waste from the wind power industry as the precursor, and its adsorption behavior towards methylene blue (MB) was systematically investigated. The maximum adsorption capacity of the as-prepared material for MB reached 1037.76 mg/g, which was significantly superior to similar biomass-based adsorbents. Efficient removal of high-concentration MB was achieved at a low adsorbent dosage, and the material maintained stable performance in a wide pH range. The adsorption process was dominated by monolayer physisorption, and the synergistic effect of pore confinement, π-π conjugation, electrostatic interaction and hydrogen bonding was the core mechanism for its efficient adsorption. This study provides a new route for the high-value utilization of balsa wood industrial solid waste, and also offers a high-performance alternative adsorbent material for dye wastewater treatment.

## Figures and Tables

**Table 1:** Langmuir, Freundlich, Temkin, and Dubinin-Radushkevich isotherm model parameters and correlation coefficients for methylene blue onto SAC at 30 °C.

Isotherm	Parameters		R^2^
Langmuir	q_m_ (mg/g)	K_L_ (L/mg)	0.9995
	1037.76	0.42	
Freundlich	1/n	K_F_ [(mg/g) (L/mg)^1/n^]	0.9635
	0.138	537.0	
Temkin	B (J/mol)	A(L/g)	0.9846
	103.13	173.3	
Dubinin-Radushkevich	q_s_ (mg/g)	E (kJ/mol)	0.7838
	932.21	8.12	

**Table 2:** Comparison of the maximum MB adsorption capacity between the as-prepared SAC and other reported biomass-based activated carbons.

Adsorbent	Maximum Monolayer Adsorption Capacity (mg/g)	References
*Ochroma pyramidale*	1037.7	This work
*Sugarcane bagasse*	1017.2	[20]
*Longan seed*	1000.0	[21]
*Tobacco straw*	849.9	[22]
*Bamboo fiber*	816.0	[23]
*Wood tar*	528.0	[24]
*Pomelo Peel*	602.4	[25]
*Mangosteen peel*	871.4	[26]

**Table 3:** Pseudo-first-order and pseudo-second-order kinetic model parameters and correlation coefficients for methylene blue onto SAC at 30 °C.

C_0_	q_e,exp_ (mg/g)	Pseudo-First-Order Kinetic Model			Pseudo-Second-Order Kinetic Model		
		q_e,cal_ (mg/g)	K_1_	R^2^	q_e,cal_ (mg/g)	K_2_	R^2^
200	499.91	7.30	0.093	0.957	500.00	3.64 × 10^−2^	0.9991
300	746.36	39.36	0.042	0.979	746.27	3.33 × 10^−3^	0.9988
400	952.44	269.08	0.063	0.877	961.54	7.02 × 10^−4^	0.9988
500	1001.75	157.95	0.032	0.989	1011.17	6.35 × 10^−4^	0.9987
600	1039.13	240.08	0.050	0.981	1053.64	5.63 × 10^−4^	0.9987

## Data Availability

The data that support the findings of this study are available within the article. Further inquiries may be addressed to the corresponding author.

## Supplementary Materials

The following supporting information can be downloaded at: https://www.mdpi.com/article/10.3390/molecules31081251/s1. The following are available online: Figure S1: Effects of adsorption time and initial concentration on MB adsorption by SAC, and the fitting methods for different adsorption isotherm models.
