# Supplementary material for: High-Efficiency Adsorption of Methylene Blue by Balsa Wood Waste-Based Microporous Carbon
Source: Molecules. 2026 Apr 9;31(8):1251. doi: 10.3390/molecules31081251 (PMC13118323; doi:10.3390/molecules31081251)
Supplement: Supplementary file 1 [file molecules-31-01251-s001.zip › molecules-4216434-supplementary.pdf]

Supporting Information

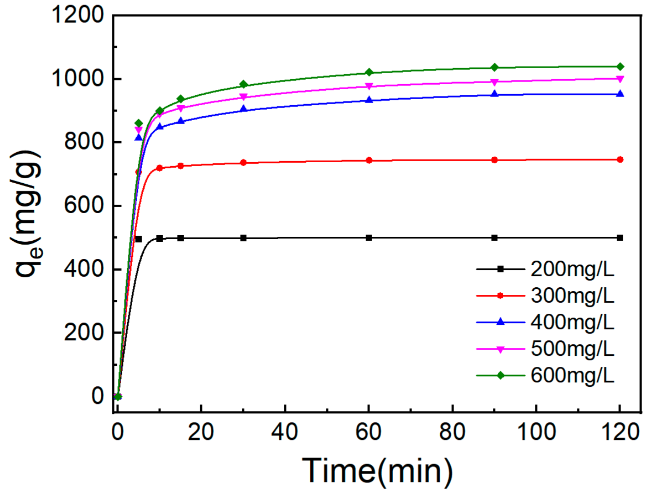

Figure S1. Effects of adsorption time and initial concentration on MB adsorption by SAC.

In the initial stage of adsorption, the adsorption capacity of SAC increased rapidly and nearly reached saturation. Thereafter, the adsorption capacity continued to increase slowly with time until equilibrium was attained. At lower initial MB concentrations (e.g., 200 or 300 mg/L), adsorption saturation was achieved within only 15 min. At higher initial concentrations (e.g., 500 or 600 mg/L), the time required to reach saturation increased to 100 min. This behavior is attributed to the abundance of active sites on the SAC and the high MB concentration in solution during the initial adsorption stage, resulting in a fast adsorption rate. As adsorption proceeds, the active sites become progressively occupied, and the MB concentration in solution decreases, leading to a gradual slowdown of the adsorption process.

1. Fitting methods for different adsorption isotherm models

1.1 Langmuir isotherm

The Langmuir isotherm assumes uniform energies of adsorption on the

surface and no transmigration of adsorbate in the plane of the surface. In other words, uptake occurs on a homogeneous surface by monolayer sorption without interaction between the adsorbed molecules. The linear form of the Langmuir isotherm is given by the following equation:

$$\frac{C_e}{q_e}=\frac{1}{q_m K_L}+\frac{1}{q_m}C_e$$

where qe is the adsorption capacity at equilibrium (mg/g), Ce is the equilibrium concentration of methylene blue (mg/L), and qm and KL are Langmuir constants related to maximum monolayer adsorption capacity and rate of adsorption, respectively. When Ce/qe is plotted against Ce, a straight line with slope 1/qm and intercept 1/qmKL is obtained

1.2 Freundlich isotherm

The Freundlich isotherm, as a fairly satisfactory empirical isotherm used for nonideal sorption, assumes heterogeneous surface energies. The equation of the Freundlich isotherm is shown by:

$$q_e=K_F C_e^{\frac{1}{n}}$$

The linear form is expressed by the following equation by taking the logarithm of both sides :

$$\ln q_e=\ln K_F+\frac{1}{n}\ln C_e$$

where qe is the adsorption capacity at equilibrium (mg/g), Ce is the equilibrium concentration of methylene blue (mg/L), and KF and n are Freundlich constants. The constant n gives an indication of how favorable the

adsorption process is while KF represents the quantity of dye adsorbed onto the adsorbent for a unit equilibrium concentration. The slope 1/n ranging from 0 to 1 is a measure of adsorption intensity or surface heterogeneity.

1.3 Temkin isotherm

Temkin and Pyzhev proposed that the effects of indirect adsorbate/adsorbate interactions on adsorption isotherms cannot be ignored. The adsorption heats of all molecules in a layer would decrease linearly as the surface is covered due to adsorbate/adsorbate interactions. Therefore, the equation for the Temkin isotherm was developed as follows:

$$q_e = \frac{RT}{b}\ln(AC_e)$$

where R is the gas constant (8.314 J/mol K), T (K) is the absolute temperature, and RT/b is denoted by B.

1.4 Dubinin–Radushkevich isotherm

Another popular equation for analyzing adsorption isotherms is the Dubinin–Radushkevich isotherm proposed by Dubinin and Radushkevich, as follows :

$$q_e = q_s \exp(-K\varepsilon^2)$$

where ε can be correlated:

$$\varepsilon = RT\ln\left(1 + \frac{1}{C_e}\right)$$

where R is the gas constant (8.314 J/mol K) and T is the absolute temperature. The linear form of the Dubinin–Radushkevich isotherm is given

by the following equation:

$$\ln q_e = \ln q_s - K\varepsilon^2$$

The adsorption free energy (E) of each adsorbate molecule can be calculated from the constant K when the adsorbate molecule is transferred to the surface of the adsorbent from infinity in the solution, as follows:

$$E = \frac{1}{\sqrt{2K}}$$

2 The Kinetic Adsorption equation

2.1 Pseudo-first-order kinetics

Adsorption kinetic data can be analyzed by the pseudo-first-order kinetic equation, given by Langergren and Svenska, as follows:

$$\frac{dq_t}{dt} = K_1(q_e - q_t)$$

where K_1 is the rate constant of the pseudo-first-order kinetic process and q_t and q_e are the adsorption capacity of methylene blue onto the SAC at time t and equilibrium, respectively. The linear form is expressed by the following equation:

$$\ln(q_e - q_t) = \ln q_e - K_1 t$$

2.2 Pseudo-second-order kinetics

The adsorption kinetic data can also be analyzed through the pseudo-second-order kinetic equation, as follows:

$$\frac{1}{q_e - q_t} = \frac{1}{q_e} + K_2 t$$

where K_2 is the rate constant of the pseudo-second-order kinetic process and q_t and q_e are the adsorption capacity of methylene blue onto SAC at time t and equilibrium, respectively. The linear form is expressed by the following equation:

$$\frac{t}{q_t} = \frac{1}{K_2 q_e^2} + \frac{1}{q_e} t$$
